# Interactions between ALDH2 rs671 polymorphism and lifestyle behaviors on coronary artery disease risk in a Chinese Han population with dyslipidemia: A guide to targeted heart health management

**DOI:** 10.1186/s12199-018-0719-y

**Published:** 2018-06-30

**Authors:** Liu Huang, Xiao Cai, Fuzhi Lian, Long Zhang, Yuling Kong, Chengjian Cao, Haiyan Ma, Yuxian Shao, Yinyin Wu, Baodan Zhang, Liangwen Xu, Lei Yang

**Affiliations:** 1 Department of Health Management, School of Medicine, Hangzhou Normal University, Zhejiang, 310036 Hangzhou China; 2 Hangzhou Hospital for the Prevention and Treatment of Occupational Diseases, Zhejiang, Hangzhou China

**Keywords:** ALDH2, Coronary artery disease, Heart health management, Lifestyle, Single nucleotide polymorphism

## Abstract

**Background:**

Both aldehyde dehydrogenase 2 (ALDH2) rs671 polymorphism and lifestyle behaviors are involved in coronary artery disease (CAD), while the interaction between them is currently unknown.

**Methods:**

A nested case-control study was conducted in 161 patients with CAD and 495 controls in dyslipidemia population in Yinzhou District, Ningbo, Zhejiang Province, China, in August 2013. Anthropometric data and blood samples were collected, demographic characteristics and lifestyle behaviors information were obtained by a face-to-face interview, dietary intake was assessed by a food frequency questionnaire, and genomic DNA was genotyped.

**Results:**

Carriers with increasing number of A alleles had an elevated CAD risk compared with G allele carriers (adjusted OR = 1.483, 95% CI = 1.114–1.974). Carriers of rs671 A/G and A/A genotypes had a higher CAD risk than carriers of G/G genotype (adjusted OR = 1.492, 95% CI = 1.036–2.148). Similarly, individuals with rs671 A/A genotype had a higher CAD risk than individuals with A/G and G/G genotypes (adjusted OR = 2.161, 95% CI = 1.139–4.101). We found a borderline additive interaction between regular fried food intake and A/A and A/G genotypes, and a significantly additive interaction between sedentary/light physical activity and A/A and A/G genotypes.

**Conclusions:**

Individuals with A/A or A/G genotypes of rs671 have a higher CAD risk, if they lack physical activity and take fried food regularly, than individuals with G/G genotypes. These findings can help to provide a guide to targeted heart health management.

**Electronic supplementary material:**

The online version of this article (10.1186/s12199-018-0719-y) contains supplementary material, which is available to authorized users.

## Background

Both genetic factors and lifestyle behaviors are crucial drivers of coronary artery disease (CAD), also known as ischaemic heart disease (IHD) [1], which is the leading cause of death worldwide and the second cause of death in China [2]. With the development of genome-wide association studies (GWAS), genetic susceptibility of CAD, identified by single nucleotide polymorphism (SNP), has become an important area of research in the postgenomic era. So far, genome-wide association analyses have identified over 66 independent CAD risk SNPs [3–8]. Among these SNPs, the Aldehyde dehydrogenase 2 (ALDH2) rs671 polymorphism has attracted increasing attention [7, 9, 10]. ALDH2 rs671 polymorphism is in exon 12 within ALDH2 gene which located on chromosome 12q24, and ALDH2 encoded by the ALDH2 gene was initially recognized as a key enzyme involved in the metabolism of alcohol (ethanol) [11]. ALDH2 rs671 polymorphism exists mainly in the East Asian population, whereas it is quite rare in Caucasian and African population [12]. Accumulating evidence suggests that the ALDH2 rs671 polymorphism is associated with the risk of CAD in Asian population [8, 13–16], and the mechanisms may relate to the effect of ALDH2 rs671 polymorphism on hypertension [17–19], dyslipidemia [13, 20, 21], and endothelial asymmetric dimethylarginine (ADMA) levels [9].

Plenty of evidence has also validated that a healthy lifestyle can dramatically reduce rates of incident cardiovascular events [22–25]. No matter in the Victoria declaration made early in 1992 or the recent guideline on heart health, the favorable lifestyle behaviors which include health-promoting dietary habits, a tobacco-free lifestyle, and regular physical activity underlie the cornerstone of heart health in the general population [26, 27]. Likewise, current findings have demonstrated that among individuals, even with a genetic predisposition to CAD, the healthy lifestyle is still associated with a decreased risk for CAD [28].

However, very few researches have directly focused on the interaction between the ALDH2 rs671 polymorphism and lifestyle behaviors on the risk of CAD. Hence, in this study we evaluated the interaction between the ALDH2 rs671 polymorphism and lifestyle behaviors on CAD on the additive scale using the relative excess risk due to interaction (RERI) with *P* values and confidence intervals, which has been often regarded as the standard measure for interaction on the additive scale in case-control studies [29–33]. The synergism, assessed by the RERI, between a certain ALDH2 rs671 polymorphism genotype and lifestyle behavior can help to provide the personalized and targeted health care management service, especially, lifestyle behavior guidance in the future.

## Methods

### Study population

We conducted a nested case-control study in a cohort of dyslipidemia subjects who participated in routine health examination at local community health centers during April and July 2013 in Yinzhou District of Ningbo, Zhejiang Province, China. Based on the electronic health records data set from Yinzhou Centers for Disease Control, we originally included 1359 unrelated participants, aged 40 to 80, who were diagnosed with dyslipidemia, and had not taken antihypertensive or cholesterol-lowering medications. Here, dyslipidemia was diagnosed in light of the Chinese guidelines on prevention and treatment of dyslipidemia 2007 version, according to which individuals with total cholesterol (TC) greater than 5.18 mmol/L, and/or triglyceride (TG) greater than 1.70 mmol/L, and/or high-density lipoprotein cholesterol (HDL-C) less than 1.04 mmol/L, and/or low-density lipoprotein cholesterol (LDL-C) greater than 3.37 mmol/L were defined as dyslipidemia.

Informed consent was obtained from all individual participants included in the study, and the study protocols were approved by the Medical Ethical Committee of the Affiliated Hospital of Hangzhou Normal University. After a 3-year follow-up period, in August 2016, 161 newly diagnosed CAD cases, and 495 control cases, matched for age and gender, were recruited from the cohort of dyslipidemia population. The CAD was confirmed according to the diagnostic criteria for coronary atherosclerotic heart disease of China, which is approved by the National Health and Family Planning Commission of the People’s Republic of China [34]. This study was limited to Han population in eastern China.

### Lifestyle behavior investigation and anthropometric data collection

When the cohort of dyslipidemia population was confirmed in 2013, the demographic characteristics, lifestyle behaviors, and medical history information was obtained using a self-made questionnaire by a face-to-face interview. The dietary intake preference was assessed by a semi-quantitative food frequency questionnaire.

In this survey, the current smoking was defined as smoking ≥ 1 cigarette per day for ≥ 1 year; the current alcohol intake was defined as drinking ≥ 500 g of beer, 150 g of wine, or 50 g of distilled spirit per day for ≥ 1 year; and the physical activity intensity was classified into four sorts, which were sedentary, light, moderate, and heavy, respectively. The sedentary-intensity physical activity was defined as activities of office staff who was doing almost no daily exercise. The light-intensity physical activity was defined as activities of waiters, salesmen, and teachers, which demanded the least amount of effort. The moderate-intensity physical activity was defined as activities which required prolonged period of walking, pushing or pulling objects. The heavy-intensity physical activity was defined as activities which involved tough effort and frequent extensive body movement, such as activities for non-mechanized farming, construction workers, dockworkers, and dancers. The frequency of fried food and dessert intake was classified into two sorts, which were never and regular intake, respectively. The frequency for the never intake was defined as ≤ once a week, and for the regular intake was defined as ≥ 1 to 4 times per week.

The anthropometric data, including waist, body mass index (BMI), systolic blood pressure (SBP), diastolic blood pressure (DBP), and TC, TG, HDL-C, LDL-C levels was assessed in the physical examination by professional staff in accordance with a standard protocol.

### Blood sample collection and SNP genotyping

5 ml peripheral blood samples were collected from the participants in both cases and controls in 2013. Genomic DNA was extracted from the peripheral blood samples using TIANamp Blood DNA Kits (Tiangen Biotech, Beijing, China) following the manufacturer’s protocol. ALDH2 rs671 polymorphism genotyping was performed using polymerase chain reaction-ligase detection reaction (PCR-LDR) method (Generay Biotech Company, Shanghai, China). Primer sequences and reaction conditions used were described previously [17]. For quality control, we randomly chose 10% of the samples to re-genotype, and the concordance was 100%.

### Statistical analysis

The analysis of demographic characteristics, as well as the interaction between ALDH2 rs671 polymorphism and lifestyle factors on the risk of CAD, was conducted in SPSS 24.0 software (SPSS Inc., Chicago, IL, USA). Specifically, the differences in variables between CAD cases and control subjects were evaluated by using Pearson chi-squared test for categorical variables expressed as frequencies (percentages, %), Student’s *t* test for normally distributed continuous variables expressed as mean (standard deviation, SD), and Mann-Whitney *U* test for non-normally distributed continuous variables expressed as median (interquartile range, IQR). The interaction between ALDH2 rs671 polymorphism and lifestyle factors on CAD was evaluated by using unconditional logistic regression under a dominant model after adjustment for potential confounders [35]. In the logistic regression analysis, the dummy variables which represented the subgroups of the ALDH2 rs671 polymorphism and lifestyle factors were created. In addition, in PLINK 1.9 software incorporated into gPLINK (version 2.050), the association of ALDH2 rs671 polymorphism with the risk of CAD was evaluated by using logistic regression after adjusting for potential confounders in three genetic models, which are additive, dominant, and recessive models respectively [36]. Finally, tests of interaction between ALDH2 rs671 polymorphism and lifestyle factors on an additive scale were conducted by using relative excess risk due to interaction (RERI), as described by Knol et al. [29, 37]. The statistical significance level was set at *P* value< 0.05 (two-sided), and the Benjamini–Hochberg procedure with a false discovery rate at 0.12 was used to adjust for multiple comparisons concerning all the 46 tests presented in this study [38].

## Results

### General characteristics of the participants recruited

Table 1 shows that CAD cases and controls did not differ with respect to age. In addition, the two study groups were similar with respect to waist, body mass index (BMI), diastolic blood pressure (DBP), total cholesterol (TC) level, high-density lipoprotein cholesterol (HDL-C) level, low-density lipoprotein cholesterol (LDL-C) level, current smoking status, current alcohol intake status, as well as the history of diabetes mellitus. Whereas, the systolic blood pressure (SBP) and total cholesterol (TC) level were higher in the controls compared with the CAD cases (145 mmHg versus 142 mmHg, *P* value = 0.029; 1.58 mmol/L versus 1.43 mmol/L, *P* value = 0.012). Besides, there were significant differences in physical activity intensity, fried food intake, dessert intake, and distribution of rs671 genotypes. These results were still significant using the Benjamini–Hochberg procedure with the false discovery rate at 0.12. Other lifestyle behavior characteristics of the participants are presented in the Additional file 1: Table S1.

**Table 1:** General characteristics of the participants recruited in the CAD cases and controls

Characteristics	CAD cases (*n* = 161)	Control subjects (*n* = 495)	*P* value
Age, median (IQR), years	68.00 (14.50)	68.00 (13.00)	0.455
Gender, *n* (%)			
Male	69 (42.9)	212 (42.8)	0.995
Female	92 (57.1)	283 (57.2)	
Current smoking, *n* (%)	36 (22.4)	98 (19.8)	0.484
Current alcohol intake, *n* (%)	35 (21.7)	134 (27.1)	0.179
Diabetes mellitus, *n* (%)	10 (6.2)	29 (5.9)	0.869
Physical activity intensity, *n* (%)			
Sedentary/light	*140 (87.0)*	*387 (78.2)*	*0.015*
Moderate/heavy	*21 (13.0)*	*108 (21.8)*	
Fried food intake, *n* (%)			
Never	*52 (32.3)*	*213 (43.0)*	*0.016*
Regular	*109 (67.7)*	*282 (57.0)*	
Dessert intake, *n* (%)			
Never	*37 (23.0)*	*172 (34.7)*	*0.005*
Regular	*124 (77.0)*	*323 (65.3)*	
Waist, median (IQR), cm	84.00 (12.00)	83.00 (11.00)	0.175
BMI, mean (SD), kg/m^2^	24.22 (3.22)	23.90 (2.95)	0.263
SBP, median (IQR), mmHg	*142.00 (20.00)*	*145.00 (26.00)*	*0.029*
DBP, median (IQR), mmHg	90.00 (14.00)	90.00 (15.00)	0.552
TC, median (IQR), mmol/L	5.37 (1.12)	5.42 (1.05)	0.241
TG, median (IQR), mmol/L	*1.43 (0.89)*	*1.58 (0.94)*	*0.012*
HDL-C, median (IQR), mmol/L	1.20 (0.44)	1.21 (0.42)	0.928
LDL-C, median (IQR), mmol/L	3.54 (1.11)	3.47 (1.19)	0.068
Rs671 genotypes, *n* (%)			
A/A	*18 (11.2)*	*27 (5.5)*	
A/G	*71 (44.1)*	*194 (39.2)*	*0.010*
G/G	*72 (44.7)*	*274 (55.3)*	

Italic values are statistically significant with *P* value < 0.05 and maintains significance using the Benjamini–Hochberg procedure with the false discovery rate at 0.12

Abbreviations: *CAD*, coronary artery disease; *BMI*, body mass index; *SBP*, systolic blood pressure; *DBP*, diastolic blood pressure; *TC*, total cholesterol; *TG*, triglyceride; *HDL-C*, high-density lipoprotein cholesterol; *LDL-C*, low-density lipoprotein cholesterol

### Association between ALDH2 rs671 polymorphism and CAD

The distribution of the ALDH2 rs671 genotypes in controls was in Hardy–Weinberg equilibrium. Table 2 demonstrates the association of ALDH2 rs671 polymorphism with the risk of CAD in three genetic models. In additive model, carriers with an increasing number of A alleles appeared to have an elevated risk for CAD compared with individuals with G alleles (adjusted OR = 1.483, 95% CI = 1.114–1.974, *P* value = 0.007). Therefore, the A allele was regarded as the risk allele for CAD, and the G allele was considered as the non-risk allele in our study. In the dominant model, carriers of the rs671 A/G and A/A genotypes tended to have a higher risk for CAD than carriers of G/G genotype (adjusted OR = 1.492, 95% CI = 1.036–2.148, *P* value = 0.032). Similarly, in the recessive model, individuals of the rs671 A/A genotype tended to have a higher risk for CAD than individuals of A/G and G/G genotypes (adjusted OR = 2.161, 95% CI = 1.139–4.101, *P* value = 0.018). All the ORs were adjusted for potential confounders including age, gender, BMI, TG level, TC level, HDL-C level, SBP, current smoking status, current alcohol intake status, and the history of diabetes mellitus. These associations maintained significance when using the Benjamini–Hochberg procedure to adjust for multiple comparisons.

**Table 2:** Associations of ALDH2 rs671 polymorphism with CAD risk in three genetic models

ALDH2 rs671	CAD cases *n* (%)	Control subjects *n* (%)	Crude OR (95% CI)	*P* value	Adjusted OR^†^ (95% CI)	*P* value
Additive model						
G	215	742	1.0		1.0	
A	107	248	*1.508 (1.141–1.994)*	*0.004*	*1.483 (1.114–1.974)*	*0.007*
Dominant model						
G/G	72	274	1.0		1.0	
A/G + A/A	89	221	*1.533 (1.072–2.192)*	*0.019*	*1.492 (1.036–2.148)*	*0.032*
Recessive model						
A/G + G/G	143	468	1.0		1.0	
A/A	18	27	*2.182 (1.168–4.077)*	*0.014*	*2.161 (1.139–4.101)*	*0.018*

^†^Adjusted for age, gender, BMI, TG level, TC level, HDL-C level, SBP, current smoking status, current alcohol intake status, and the history of diabetes mellitus

Italic values are statistically significant with *P* value < 0.05 and maintains significance using the Benjamini–Hochberg procedure with the false discovery rate at 0.12

### Interaction between ALDH2 rs671 polymorphism and dessert intake on CAD

Table 3 indicates the joint analysis on the interaction between ALDH2 rs671 polymorphism and dessert intake on the CAD risk. After adjusting for potential confounders, compared with individuals with G/G genotype and no dessert intake preference, individuals with G/G genotype and regular dessert intake preference, as well as individuals with A/A and A/G genotypes and regular dessert intake preference had an elevated CAD risk (adjusted OR = 2.095, 95% CI = 1.113–3.944, *P* value = 0.022; adjusted OR = 2.539, 95% CI = 1.335–4.829, *P* value = 0.004, respectively). Within the A/A and A/G genotypes stratum, regular dessert intake did not significantly increased the risk for CAD, compared with no dessert intake. No significantly positive interaction between the A/A and A/G genotypes and the regular dessert intake was found on the additive scale.

**Table 3:** Interaction between ALDH2 rs671 polymorphism and dessert intake on CAD risk

	ALDH2 rs671 polymorphism genotype				
	Non-risk allele carriers (G/G)		Risk allele carriers (A/A + A/G)		OR^†^ (95% CI) for A/A + G/G within strata of dessert intake
	*N* cases/controls	OR^†^ (95% CI)	*N* cases/controls	OR^†^ (95% CI)	
No dessert intake	15/101		22/71		
		1.0		1.950 (0.930–4.088) *P* = 0.077	1.950 (0.930–4.088) *P* = 0.077
Regular dessert intake	57/173		67/150		
		*2.095 (1.113–3.944)* *P = 0.022*		*2.539 (1.335–4.829)* *P = 0.004*	1.212 (0.773–1.901) *P* = 0.402
OR^†^ (95% CI) for regular dessert intake within strata of genotype		*2.095 (1.113–3.944)* *P = 0.022*		1.302 (0.727–2.331) *P* = 0.374	

Measure of interaction on additive scale: RERI (95% CI) = − 0.506 (− 2.110 to 1.099); *P* value = 0.537

^†^Adjusted for age, sex, BMI, SBP, TC level, TG level, HDL-C level, current smoking, current alcohol intake, and history of diabetes mellitus

Italic values are statistically significant with *P* value < 0.05 and maintains significance using the Benjamini–Hochberg procedure with the false discovery rate at 0.12

### Interaction between ALDH2 rs671 polymorphism and fried food intake on CAD

Table 4 presents the interaction between ALDH2 rs671 polymorphism and fried food intake on the risk for CAD. After adjustment for potential confounders, compared with individuals with G/G genotype and no fried food intake preference, there was an increased CAD risk among individuals with both A/A and A/G genotypes and regular fried food intake preference (adjusted OR = 1.859, 95% CI = 1.074–3.217, *P* value = 0.027). Within the A/A and A/G genotypes stratum, regular fried food intake was associated with an elevated risk for CAD, compared with no fried food intake (adjusted OR = 1.949, 95% CI = 1.117–3.400, *P* value = 0.019). In addition, within the regular fried food intake stratum, carriers with A/A and A/G genotypes had an increased risk for CAD, compared with carriers with G/G genotype (adjusted OR = 1.715, 95% CI = 1.057–2.780, *P* value = 0.029). These associations remained significant by the Benjamini–Hochberg procedure. Borderline interaction between the A/A and A/G genotypes and regular fried food intake was found (RERI = 0.821, 95% CI = − 0.007 to 1.649, *P* value = 0.052).

**Table 4:** Interaction between ALDH2 rs671 polymorphism and fried food intake on CAD risk

	ALDH2 rs671 polymorphism genotype				
	Non-risk allele carriers (G/G)		Risk allele carriers (A/A + A/G)		
	N cases/controls	OR^†^ (95% CI)	*N* cases/controls	OR^†^ (95% CI)	OR^†^ (95% CI) for A/A + G/G within strata of fried food intake
No fried food intake	28/116		24/97		
		1.0		0.954 (0.509–1.786) *P* = 0.882	0.954 (0.509–1.786) *P* = 0.882
Regular fried food intake	44/158		65/124		
		1.084 (0.626–1.878) *P* = 0.773		*1.859 (1.074–3.217)* *P = 0.027*	*1.715 (1.057–2.780)* *P = 0.029*
OR^†^ (95% CI) for regular fried food intake within strata of genotype		1.084 (0.626–1.878) *P* = 0.773		*1.949 (1.117–3.400)* *P = 0.019*	

Measure of interaction on additive scale: RERI (95% CI) = 0.821 (−0.007 to 1.649); *P* value = 0.052

^†^Adjusted for age, sex, BMI, SBP, TC level, TG level, HDL-C level, current smoking, current alcohol intake, and history of diabetes mellitus

Italic values are statistically significant with *P* value < 0.05 and maintains significance using the Benjamini–Hochberg procedure with the false discovery rate at 0.12

### Interaction between ALDH2 rs671 polymorphism and physical activity on CAD

Table 5 shows the interaction between ALDH2 rs671 polymorphism and physical activity intensity on the CAD risk. After adjustment for potential confounders, compared with individuals with G/G genotype and moderate/heavy physical activity, there was an elevated risk for CAD among individuals with both A/A and A/G genotypes and sedentary/light physical activity (adjusted OR = 2.071, 95% CI = 1.013–4.234, *P* value = 0.046). Within the A/A and A/G genotypes stratum, sedentary/light physical activity conferred an increased risk of CAD, compared with the moderate/heavy physical activity (adjusted OR = 2.612, 95% CI = 1.202–5.674, *P* value = 0.015). Besides, within the sedentary/light physical activity stratum, individuals with the A/A and A/G genotypes had a higher CAD risk than those with the G/G genotype (adjusted OR = 1.540, 95% CI = 1.012–2.344, *P* value = 0.044). Significantly positive interaction between the A/A and A/G genotypes and the sedentary/light physical activity was found (RERI = 0.933, 95% CI = 0.021 to 1.845, *P* value = 0.045). These associations maintained significance using the Benjamini–Hochberg correction for multiple comparisons.

**Table 5:** Interaction between ALDH2 rs671 polymorphism and physical activity on CAD risk

	ALDH2 rs671 polymorphism genotype				
	Non-risk allele carriers (G/G)		Risk allele carriers (A/A + A/G)		OR^†^ (95% CI) for A/A + G/G within strata of physical activity
	*N* cases/controls	OR^†^ (95% CI)	*N* cases/controls	OR^†^ (95% CI)	
Moderate/heavy physical activity	12/59		9/49		
		1.0		0.793 (0.299–2.101) *P* = 0.640	0.793 (0.299–2.101) *P* = 0.640
Sedentary/light physical activity	60/215		80/172		
		1.345 (0.669–2.701) *P* = 0.406		*2.071 (1.013–4.234)* *P = 0.046*	*1.540 (1.012–2.344)*
					*P = 0.044*
OR^†^ (95% CI) for sedentary/light physical activity within strata of genotype		1.345 (0.669–2.701) *P* = 0.406		*2.612 (1.202–5.674)* *P = 0.015*	

Measure of interaction on additive scale: RERI (95% CI) = 0.933 (0.021 to 1.845); *P value = 0.045*

^†^Adjusted for age, sex, BMI, SBP, TC level, TG level, HDL-C level, current smoking, current alcohol intake, and history of diabetes mellitus

Italic values are statistically significant with *P* value < 0.05 and maintains significance using the Benjamini–Hochberg procedure with the false discovery rate at 0.12

## Discussion

CAD is inevitably affected by both genetic and lifestyle factors; therefore, the interaction between these two factors is well worth evaluating. Assessing their interaction on CAD risk can help to provide targeted lifestyle guidance for the individuals with different ALDH2 rs671 polymorphism genotypes. Nevertheless, there has not been yet any study on this issue.

In this nested case-control study, we found the association between ALDH2 rs671 polymorphism and CAD risk. Besides, the frequency for dessert intake, for fried food intake, and physical activity intensity were different between the CAD cases and the controls. In addition, we found a significantly positive interaction between the A/A and A/G rs671 genotypes and the sedentary/light physical activity, as well as a borderline interplay between the A/A and A/G rs671 genotypes and regular fried food intake.

The result of the association between ALDH2 rs671 polymorphism and an elevated CAD risk was consistent with findings from previous studies in China [9, 10] and Japan [7]. Such findings support our concern about the increased CAD risk resulting from ALDH2 rs671 polymorphism in Han population in eastern China. Besides, the differences of the dessert intake frequency, fried food intake frequency, and physical activity levels suggested that these lifestyle behaviors might affect the CAD risk in this population, which are partly supported by the prospective study in the US [39]. Furthermore, the RERI of the A/A and A/G rs671 genotypes and the sedentary/light physical activity level indicated a significant synergism between them, and the RERI of the A/A and A/G rs671 genotypes and the regular fried food intake suggested a borderline synergism between them. For heart health purposes, RERI, as a measure of the interaction on the additive scale and synergism, suggests whether the impact of a risk factor would be greater in one certain subpopulation than in another, and is hence of great importance in identifying specific high-risk individuals and in lifestyle guidance [29]. Therefore, according to our findings, we shall suggest that individuals with the A/A and A/G genotypes of ALDH2 rs671 polymorphism should adhere to an intensive physical activity and take less fried food than individuals with G/G genotype. These results can be applied to the development of heart health promotion strategies especially in the East Asian, as the ALDH2 rs671 polymorphism existed mainly in the East Asian population [12].

Interestingly, in our research, the regular dessert intake behavior showed an increased CAD risk in carriers with non-risk G/G genotype of rs671, rather than in those with risk A/A and A/G genotypes. This result seems to suggest that the individuals only with non-risk G/G genotype of rs671 should reduce the dessert intake. Nevertheless, we think the effect of the regular dessert intake on CAD risk should be validated in a larger population, as the number of the carriers with A/A and A/G genotypes in our study is relatively small. Also, the total amount of the dessert intake should be taken into consideration, as we only estimated the frequency of the dessert intake.

Our study has several limitations. First, we only enrolled the participants with dyslipidemia in Han population in eastern China, the generalizability of our findings should be validated in more diverse populations and populations without dyslipidemia, as China is a multiethnic country and the dyslipidemia itself is a risk factor for CAD. Second, because the ALDH2 rs671 polymorphism information and the lifestyle behaviors of the participants were not randomized, the association of the interaction between ALDH2 rs671 polymorphism and the lifestyle behaviors with CAD risk cannot be considered as a causal relationship. Third, as our study has not considered the total calorie, lipid, and carbohydrate intake or the total amount of the dessert and fried food intake, the interaction between ALDH2 rs671 polymorphism and these factors was not able to be analyzed. Fourth, the sample size of this study was relatively small. As a result, we could not further divide the participants into more strata, according to the four levels of intensity of physical activity or the specific diet intake frequencies, which we focused on. Also, we could not, therefore, provide more specific lifestyle behavior guidance for individuals with different ALDH2 rs671 polymorphism genotypes. Finally, the underlying mechanism of the interaction between ALDH2 rs671 polymorphism and lifestyle behaviors on the CAD risk was not explained. We may infer, according to our current knowledge, the mechanism underlying the effect of this interaction would be very complicated; therefore, we are planning to address this complex mechanism in our future study.

## Conclusion

Individuals with A/A and A/G genotypes of ALDH2 rs671 polymorphism were confronted with a higher risk for CAD, if they were lack of physical activity and take fried food regularly, compared with individuals with G/G genotypes. These findings can help to provide a guide to the targeted heart health management strategies for the Chinese Han population with dyslipidemia.

## Additional file

Additional file 1: **Table S1** Lifestyle behavior characteristics of the participants in the CAD cases and controls. (DOC 71 kb)

## Abbreviations

ALDH2: Aldehyde dehydrogenase 2
BMI: Body mass index
CAD: Coronary artery disease
DBP: Diastolic blood pressure
GWAS: Genome-wide association study
HDL-C: High-density lipoprotein cholesterol
IHD: Ischaemic heart disease
LDL-C: Low-density lipoprotein cholesterol
PCR-LDR: Polymerase chain reaction-ligase detection reaction
RERI: Relative excess risk due to interaction
SBP: Systolic blood pressure
SNP: Single nucleotide polymorphism
TC: Total cholesterol
TG: Triglyceride
