# Supplementary material for: Interactions between ALDH2 rs671 polymorphism and lifestyle behaviors on coronary artery disease risk in a Chinese Han population with dyslipidemia: A guide to targeted heart health management
Source: Environ Health Prev Med. 2018 Jun 30;23:29. doi: 10.1186/s12199-018-0719-y (PMC6026513; doi:10.1186/s12199-018-0719-y)
Supplement: Supplementary file 1 — Table S1 Lifestyle behavior characteristics of the participants in the CAD cases and controls. (DOC 71 kb) [file 12199_2018_719_MOESM1_ESM.doc]

**Table S1** Lifestyle behaviour characteristics of the participants in the CAD cases and controls

| Characteristics | | CAD cases  (n = 161) | Control subjects (n = 495) | *P* value | |
| --- | --- | --- | --- | --- | --- |
| Vegetable intake, n (%) | |  |  |  |  |
| ＜50g/day |  | 27 (16.8) | 98 (19.8) | 0.370 |  |
| 50-150g/day |  | 111 (68.9) | 311 (62.8) |  |
| ≥150g/day |  | 23 (14.3) | 86 (17.4) |  |
| Fruit intake, n (%) | |  |  |  |  |
| ＜50g/day |  | 50 (31.1) | 189 (38.2) | 0.104 |  |
| 50-150g/day |  | 99 (61.5) | 257 (51.9) |  |
| ≥150g/day |  | 12 (7.4) | 49 (9.9) |  |
| Milk consumption, n (%) | |  |  |  |  |
| ＜200ml/day |  | 95 (59.0) | 332 (67.1) | 0.062 |  |
| ≥200ml/day |  | 66 (41.0) | 163 (32.9) |  |
| Whole egg intake, n (%) | |  |  |  |  |
| Never/week |  | 53 (32.9) | 185 (37.4) | 0.528 |  |
| 1-4/week |  | 89 (55.3) | 249 (50.3) |  |
| ≥4/week |  | 19 (11.8) | 61 (12.3) |  |
| Meat intake, n (%) | |  |  |  |  |
| ＜100g/week |  | 95 (59.0) | 315 (63.6) | 0.410 |  |
| 100-200g/week |  | 51 (31.7) | 130 (26.3) |  |
| ≥200g/week |  | 15 (9.3) | 50 (10.1) |  |
| Fish intake, n (%) | |  |  |  |  |
| ＜100g/week |  | 90 (55.9) | 288 (58.2) | 0.776 |  |
| 100-200g/week |  | 44 (27.3) | 135 (27.3) |  |
| ≥200g/week |  | 27 (16.8) | 72 (14.5) |  |
| Salt consumption, n (%) | |  |  |  |  |
| ＜6g/day |  | 109 (67.7) | 301 (60.8) | 0.179 |  |
| 6-9g/day |  | 46 (28.6) | 160 (32.3) |  |
| ≥9g/day |  | 6 ( 3.7) | 34 ( 6.9) |  |
| Exercise, n (%) | |  |  |  |  |
| ＜3 times/month | | 95 (59.0) | 294 (59.4) | 0.931 |  |
| ≥3 times/month | | 66 (41.0) | 201 (40.6) |  |
